# Lessons from a large national "hospital at home" program,

**DOI:** 10.1186/s13584-025-00741-0

**Published:** 2025-12-22

**Authors:** Alexander Lustman, Talish Razi, Maya Lerner Shikory, Naama Katz, Ruth Baruch, Shlomit Yaron, Ronen Arbel, Doron Netzer

**Affiliations:** 1 Clalit Health Services, Tel Aviv, Israel; 2 Department of Family Medicine. The Faculty of Medical and Health Sciences, Tel Aviv University, Tel Aviv, Israel; 3 Schneider Children’s Medical Center, Petach Tikvah, Israel; 4 Sapir College, Sderot, Israel; 5 Adelson School of Medicine, Ariel University, Ariel, Israel

## Abstract

**Background:**

Hospital at Home (HAH) is a potential solution to the increasing demand for hospital beds, but concerns remain about its scalability. This study examines safety, effectiveness, and patient satisfaction in a large-scale HAH program.

**Methods:**

This retrospective cohort study utilized data from Clalit Health Services (CHS). The study population included all patients participating in the HAH program during 2022 who were discharged with a primary diagnosis of pneumonia, congestive heart failure, urinary tract infection, or cellulitis. These individuals were matched with patients admitted to general medical wards, and logistic regression analysis was performed to evaluate the association between admission type and outcomes. The primary safety endpoint was all-cause mortality at 30 days, while the primary effectiveness endpoint was rehospitalization within 30 days. Patient experience was measured using a telephone questionnaire.

**Results:**

A total of 3,335 HAH patients were matched to 3,335 hospital patients. At 30 days, mortality was 192 (5.8%) for HAH patients and 305 (9.1%) for hospital patients, with an adjusted odds ratio of 0.6 (CI 0.49–0.73, P < 0.001). Readmissions at 30 days were 435 (13%) among HAH patients and 526 (16%) in hospital patients, adjusted OR 0.8 (CI 0.70–0.92, p = 0.002). 84% of patients indicated a preference for HAH over hospital admission for future care.

**Conclusions:**

HAH can provide a safe and effective setting to treat patients who need hospital-level care, with high levels of patient satisfaction. HAH has the potential to provide a scalable solution for the ever-increasing demand for hospital beds.

**Trial registration** The study was approved by the CHS (community) institutional ethics and data utilization committee (0169–21).

## Background

In many developed countries, healthcare systems grapple with increasing healthcare needs and a lack of hospital beds. Hospital at Home (HAH) provides acute-hospital-level care in the patient’s home as an alternative to treatment in a hospital medical ward [1, 2]. HAH provides comprehensive and continuous care for acute illness, including medical, nursing, paramedical, laboratory, radiology, and pharmacy care[3, 4]. Complications associated with traditional inpatient care, especially among elderly patients, are well documented [5, 6]. The rising demands placed on hospitals has also been associated with an increase in morbidity and mortality[7–10].

Studies show HAH can lower healthcare costs, improve patient satisfaction, and maintain or even enhance the quality of care provided to acutely ill adults who require hospital-level care [11–16].

Large observational studies have assessed the safety of HAH programs; however, these investigations did not include comparative analyses with patients receiving equivalent care in hospital settings [17, 18]. A cohort study involving 876 patients enrolled in virtual home care with access to urgent care visits as needed, showed there was no significant increase in mortality observed compared to hospitalized patients [19]. Similar results were found in a study of 106 HAH patients with infectious diseases [20]. A Cochrane analysis of 10 RCTs concluded that when compared to in-hospital care, HAH services may reduce 6-month mortality [21] Studies.

comparing heart failure patients treated with Hospital at Home (HAH) care and those admitted to hospital shows no significant difference in mortality or readmission rates [22–24]. A review article of patients with chronic diseases who were admitted to hospital-at-home programs via the emergency room showed patients had similar mortality risk to hospitalized patients, a 26 percent lower readmission risk, and a lower risk for admission into long-term care than their in-hospital counterparts [25]. The reviews are based on small RCTs, sometimes with limited follow-up after discharge. The lack of research on large patient samples has led to the scalability of HAH being questioned [2].

In Israel, government funding was provided to the HMOs to encourage the use of HAH from 2019until 2024 and in 2022, there were over 15,000 admissions to HAH in Clalit Health Services (CHS).

## Objective

This study evaluates the safety, effectiveness, and patient satisfaction of large-scale HAH versus traditional hospital care for patients with similar diagnoses.

## Design

### Setting

This observational, retrospective cohort study was based on data obtained from the electronic medical records of (CHS), which insures 4.78 million patients (about two-thirds of the elderly population). CHS pools the data from its many operational systems into a unified central data warehouse used for policy and research. This data repository includes detailed primary and secondary care information on hospitalizations, medications, laboratory results, and imaging test [26].

Referrals to Hospital at Home (HAH) originate from the family practitioner, emergency room, or hospital medical ward. Prior to admission, referrals are reviewed by experienced practitioners, including nurses and doctors, to assess whether the patient requires hospital-level care that can be provided in a home setting. Patients admitted to HAH receive daily visits from both physicians and nurses and have access to a 24/7 telephone line. Blood tests are performed at home, while imaging procedures take place in community clinics. Treatments delivered include intravenous medications, oxygen therapy, and nebulizer therapy. Care is provided by teams from CHS, private providers, as well as a hospital provider. In 2022, CHS recorded 15,589 admissions to HAH. 

### Study population

This study included patients over 18 years old, admitted to the HAH program during 2022, with the primary diagnosis at discharge being pneumonia, Congestive heart failure (CHF), urinary tract infection (UTI) or cellulitis. These diagnoses were the most common in HAH in 2022 and allowed a large study population that was easily comparable to hospital discharge diagnoses. Where a patient was admitted more than once during 2022, only the first admission was considered for analysis and further admissions were excluded. Exclusion criteria for the HAH group included a diagnosis of COVID-19 or pregnancy. Only patients admitted to a general medical ward were included in the analysis, while those admitted directly to high dependency wards were excluded.

### Data extraction

The following data were extracted for each participant: age at admission, sex, population sector classified into general Jewish, Arab, and Jewish ultra-Orthodox, score for socio-economic status (SES) (scores range from 1 [lowest] to 10 [highest]) and primary diagnosis at discharge. SES was based on the small statistical areas (SSA) used in the 2008 Israeli census. SSAs contain 3000–4000 people and are created to maintain homogeneity in terms of the sociodemographic composition of the population [27]. The Israeli Central Bureau of Statistics utilized demography, education, employment, housing conditions, and income to define the SSAs, and these were grouped into 20 categories. This data was updated by the POINTS Location Intelligence Company (2) [28]to improve the accuracy of the SES measure, using up-to-date sociodemographic, commercial, and housing data [29]. The entire CHS population was grouped into ten categories, ranging from 1 (lowest) to 10 (highest). In addition, we retrieved information on each patient’s co-morbidities prior to admission, including CHF, diabetes mellitus (DM), hypertension, malignancy, ischemic heart disease (IHD), smoking, obesity, and Charlson score without age [30]. Data on patient satisfaction was collected from a telephone questionnaire that included a random sample of 433 patients treated in HAH.

### Statistical analysis

The analysis was done in two stages:

First, patients admitted to HAH were matched 1:1 with patients admitted to hospital medical wards. Individual matching of patients was conducted based on primary diagnosis at discharge, sex, age (by year), and Charlson Score. Descriptive statistics were used to characterize the study participants.

In the second stage, logistic regression analysis was used to assess the association between the type of admission and outcomes adjusting for age, sex, social sector, obesity, smoking status, Charlson score (without age), and socio-economic status.

The primary endpoints were all-cause mortality and readmission at 30 days.

Kaplan–Meier curves were generated for 90-day mortality and readmission.

Analyses were conducted using R statistical software version 4.0.1 (R Project for Statistical Computing). All reported p-values are two-tailed.

## Results

### Patient population

Overall, 3,878 patients admitted to HAH met the study criteria. We were able to match 3,335 HAH of them to hospitalized patients.

Table 1 details the patient characteristics of the matched patients. The median age was 79, with 55.2% female and no difference between the groups (p > 0.9). Patients in the in-hospital care group were more likely to be from the Arab sector compared to HAH, whereas patients in HAH had a higher proportion of ultra-orthodox(p < 0.001). The socio-economic score was lower among in-hospital patients (5.0 vs 6.0, p < 0.001). A higher proportion of HAH patients were obese (48% v 45%, p = 0.02), whereas hospitalized patients were more likely to be smokers (44% v 41%, p = 0.008) or to have CHF (22% v 19%, p = 0.004). Although the point estimate of the median duration of hospitalization was similar (4.0), the 75% percentile was significantly higher in the hospitalized group compared to the HaH group (7.0 versus 5.0).

**Table 1:** Patient Characteristics

Characteristic	Overall, N = 6,670^*1*^	Hospital, N = 3,335	95% CI^*2*^	Hospital at Home, N = 3,335	95% CI^*2*^	p-value^*3*^
**Main diagnose, n, (%)**						> 0.9
CELLULITIS	1,996, (29.9%)	998, (29.9%)	28%, 32%	998, (29.9%)	28%, 32%	
CHF	768, (11.5%)	384, (11.5%)	10%, 13%	384, (11.5%)	10%, 13%	
PNEUMONIA	1,732, (26.0%)	866, (26.0%)	24%, 27%	866, (26.0%)	24%, 27%	
URINARY INFECTION	2,174, (32.6%)	1,087, (32.6%)	31%, 34%	1,087, (32.6%)	31%, 34%	
**Sex, n, (%)**						> 0.9
Male	2,990, (44.8%)	1,495, (44.8%)	43%, 47%	1,495, (44.8%)	43%, 47%	
Female	3,680, (55.2%)	1,840, (55.2%)	53%, 57%	1,840, (55.2%)	53%, 57%	
**Age at hospitalization, Median (IQR)**	79 (68, 87)	79 (68, 87)	75, 76	79 (68, 87)	75, 76	> 0.9
**Sector, n (%)**						< 0.001
General Jewish	5,514 (83%)	2,764 (83%)	82%, 84%	2,750 (82%)	81%, 84%	
Arabic	800 (12%)	466 (14%)	13%, 15%	334 (10%)	9.0%, 11%	
Ultra-orthodox	356 (5.3%)	105 (3.1%)	2.6%, 3.8%	251 (7.5%)	6.7%, 8.5%	
**Socioeconomic score, Median (IQR)**	6.00 (4.00, 7.00)	5.00 (4.00, 7.00)	5.5, 5.6	6.00 (4.00, 7.00)	5.7, 5.8	0.001
**Hospitalization_days, Median (IQR)**	4.0 (3.0, 6.0)	4.0 (3.0, 7.0)	6.7, 7.6	4.0 (3.0, 5.0)	3.9, 4.2	0.001
**CHF, n (%)**	1,393 (21%)	744 (22%)	21%, 24%	649 (19%)	18%, 21%	0.004
**Diabetes, n (%)**	2,845 (43%)	1,439 (43%)	41%, 45%	1,406 (42%)	40%, 44%	0.4
**Malignancy, n (%)**	1,679 (25%)	829 (25%)	23%, 26%	850 (25%)	24%, 27%	0.6
**Obesity, n (%)**	3,127 (47%)	1,516 (45%)	44%, 47%	1,611 (48%)	47%, 50%	0.02
**Smoking, n (%)**	2,827 (42%)	1,467 (44%)	42%, 46%	1,360 (41%)	39%, 42%	0.008
**IHD, n (%)**	2,258 (34%)	1,132 (34%)	32%, 36%	1,126 (34%)	32%, 35%	0.9
**Hypertension, n (%)**	4,673 (70%)	2,328 (70%)	68%, 71%	2,345 (70%)	69%, 72%	0.6
**Carlson Score Without Age, Median,N**	3.00,6,670	3.00,3,335	3.7, 3.9	3.00,3,335	3.7, 3.9	> 0.9

^*1*^ n, (%); Median (IQR); n (%); Median,N

^*2*^ CI = Confidence Interval

^*3*^ Pearson’s Chi-squared test; Wilcoxon rank sum test

### Clinical outcomes

At 30 days, the mortality among in-hospital patients was 305 (9.1%) compared to 192 (5.8%) amongst HAH patients (p < 0.001), Table 2. At 90 days, mortality had increased to 541 (16%) among hospitalized patients compared to 341 (10%) in HAH (p < 0.001). The odds ratio (OR) for mortality at 30 days was 0.6 (p < 0.001), comparing HAH to hospitalized patients. Additional adjustments for the primary diagnosis and patient characteristics did not change the adjusted OR: 0.6 (p < 0.001), Table 3.

**Table 2:** Events

Characteristic	Overall, N = 6,670^*1*^	Hospital, N = 3,335	Hospital at home, N = 3,335	p-value^*2*^
**30 days readmission, n (%)**	961 (14%)	526 (16%)	435 (13%)	0.002
**90 days readmission, n (%)**	1,673 (25%)	881 (26%)	792 (24%)	0.012
**30-day mortality, n (%)**	497 (7.5%)	305 (9.1%)	192 (5.8%)	< 0.001
**90-day mortality, n (%)**	882 (13%)	541 (16%)	341 (10%)	< 0.001

^*1*^ n (%)

^*2*^ Pearson’s Chi-squared test

**Table 3:** Regression- comparison of HAH to hospitalized groups

Characteristic	30 days readmission			90 days readmission			30-day mortality			90-day mortality		
N = 6,670	OR^*1*^	95% CI^*1*^	p-value	OR^*1*^	95% CI^*1*^	p-value	OR^*1*^	95% CI^*1*^	p-value	OR^*1*^	95% CI^*1*^	p-value
**Type of hospitalization**	0.8	0.70, 0.92	0.002	0.87	0.77, 0.97	0.015	0.6	0.49, 0.73	< 0.001	0.57	0.49, 0.67	< 0.001
**Main diagnose**												
CELLULITIS	—	—		—	—		—	—		—	—	
CHF	1.28	1.01, 1.61	0.043	1.35	1.11, 1.63	0.003	2.22	1.57, 3.16	< 0.001	1.7	1.32, 2.20	< 0.001
PNEUMONIA	1.02	0.84, 1.24	0.8	0.97	0.82, 1.13	0.7	3.3	2.47, 4.48	< 0.001	2.19	1.77, 2.71	< 0.001
URINARY INFECTION	1.13	0.95, 1.36	0.2	1.12	0.96, 1.30	0.14	1.28	0.93, 1.78	0.14	1.02	0.81, 1.28	0.9
**Sex**	0.98	0.85, 1.14	0.8	1.02	0.91, 1.15	0.7	0.9	0.73, 1.11	0.3	1	0.85, 1.18	> 0.9
**Age at hospitalization**	1.01	1.00, 1.01	0.004	1.01	1.01, 1.02	< 0.001	1.08	1.07, 1.09	< 0.001	1.06	1.06, 1.07	< 0.001
**Sector**												
General Jewish	—	—		—	—		—	—		—	—	
Arabic	0.93	0.73, 1.18	0.6	0.92	0.75, 1.12	0.4	1.26	0.87, 1.81	0.2	1.13	0.85, 1.49	0.4
Ultra-orthodox	0.92	0.65, 1.28	0.6	0.94	0.72, 1.22	0.7	0.67	0.36, 1.13	0.2	0.76	0.50, 1.13	0.2
**Socioeconomic score**	0.98	0.94, 1.01	0.2	0.95	0.92, 0.98	< 0.001	0.99	0.94, 1.04	0.6	0.97	0.94, 1.01	0.2
**Obesity**	0.96	0.83, 1.11	0.6	1.13	1.01, 1.27	0.041	0.76	0.62, 0.93	0.009	0.71	0.61, 0.83	< 0.001
**Smoking**	1.02	0.88, 1.18	0.8	1	0.88, 1.13	> 0.9	1.1	0.89, 1.35	0.4	1.03	0.87, 1.21	0.7
**Charlson Score Without Age**	1.09	1.06, 1.12	< 0.001	1.11	1.08, 1.13	< 0.001	1.04	1.01, 1.08	0.023	1.09	1.06, 1.12	< 0.001

^*1*^ OR = Odds Ratio, CI = Confidence Interval

**Fig. 1:**
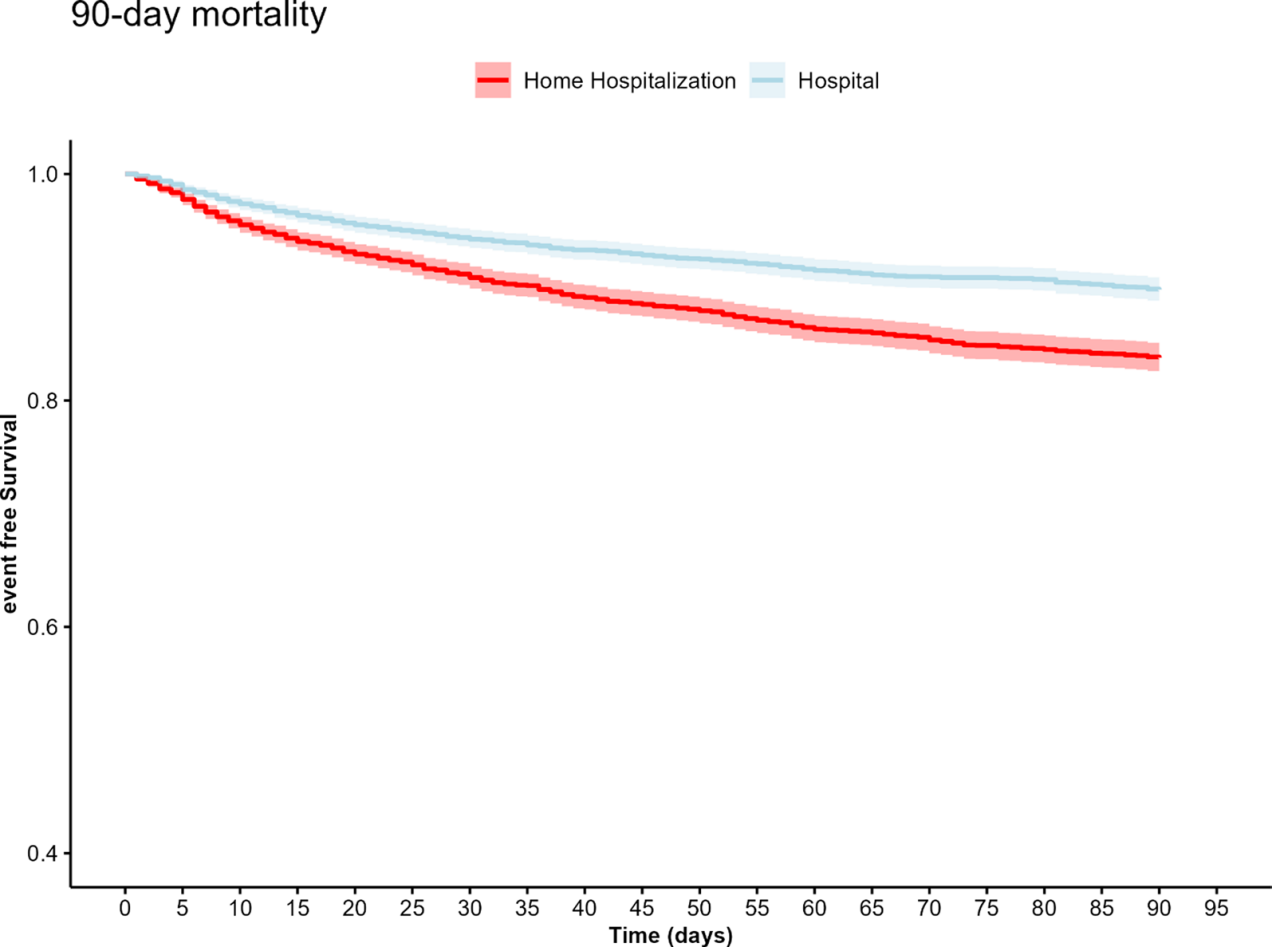
Kaplan–Meier, 90-day mortality for hospital and HAH patients

The Kaplan Meier curve for mortality shows the curves separate after about 5 days, indicating higher mortality in the hospitalized group increased risk in the hospitalized group after 5 days, which increases throughout the 90-day follow-up, Fig. 1.

**Fig. 2:**
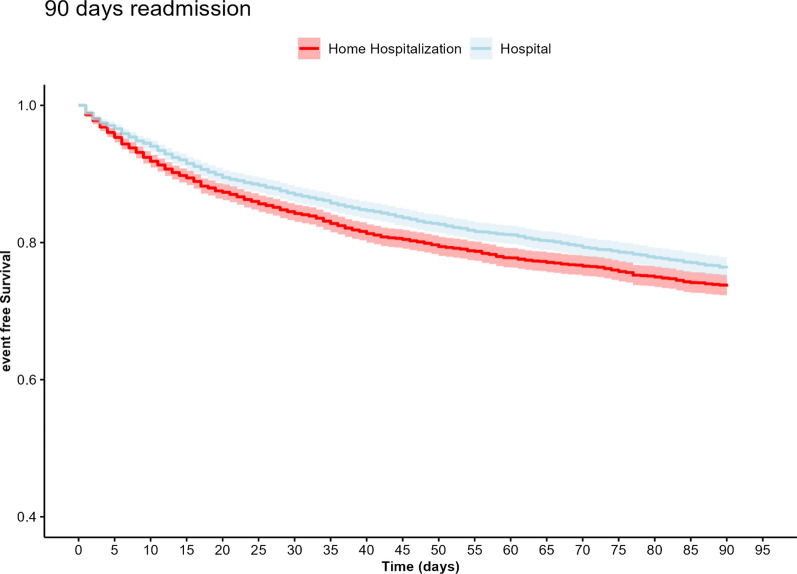
Kaplan–Meier, 90-day readmission for hospital and HAH patients

Readmissions among HAH hospitalized patients at 30 days were lower compared to hospitalized patients: 435 (13%) versus 526 (16%), adjusted OR 0.8 (p = 0.002). At 90 days, readmissions remained lower in the HAH group than among hospitalized patients, 792 (24%) versus 881 (26%) adjusted OR 0.87 (p = 0.015). The Kaplan Meier curve for readmission shows an increasing risk amongst the hospital group up to 30 days. The gap between the groups remained steady for 90 days, as depicted in Fig. 2.

When examining 30-day mortality for each of the primary diagnoses, HAH patients with a primary diagnosis of pneumonia had an adjusted OR = 0.6 (p < 0.001), UTI 0.5 (p < 0.001) and cellulitis 0.5 (p < 0.026), and the OR remained little changed for 90-day mortality. Among patients with a primary diagnosis of CHF, no significant difference was seen in mortality at 30 days OR = 1.03 (p = 0.9), or 90 days OR 0.89 (p = 0.5).

The adjusted OR for readmissions at 30 days was significantly lower amongst HAH patients with a primary diagnosis of pneumonia, OR = 0.7 (p = 0.014), UTI, OR = 0.7 (p = 0.004). There was little change in OR after 90 days for pneumonia, however the OR for readmission for UTI became non-significant (OR 0.84, p 0.082). Patients with a primary diagnosis of CHF had an adjusted OR for readmission at 30 days of 1.24 (p = 0.2) and at 90 days 1.33 (p = 0.065), these results were not statistically significant. The odds ratio for readmission for patients with cellulitis was 0.86 (p = 0.3) at 30 days and 0.83 (p = 0.11) at 90 days, Table 4.

**Table 4:** Odds Ratios comparing HAH to hospital admissions by diagnosis

	N	30 days readmission			90 days readmission			30-day mortality			90-day mortality		
		OR	95% CI	p-value	OR	95% CI	p-value	OR	95% CI	p-value	OR	95% CI	**p-value**
**PNEUMONIA**	1,732	0.7	0.53, 0.93	0.014	0.73	0.58, 0.92	0.007	0.6	0.41, 0.74	< 0.001	0.52	0.41, 0.67	** <** 0.001
**UTI**	2,174	0.7	0.55, 0.89	0.004	0.84	0.69, 1.02	0.082	0.5	0.33, 0.75	< 0.001	0.52	0.38, 0.69	< 0.001
**CHF**	768	1.24	0.86, 1.79	0.2	1.33	0.98, 1.80	0.065	1.03	0.65, 1.61	> 0.9	0.89	0.62, 1.27	0.5
**CELLULITIS**	1,996	0.86	0.65, 1.13	0.3	0.83	0.67, 1.04	0.11	0.5	0.31, 0.92	0.026	0.5	0.35, 0.72	< 0.001

Adjusted for, sex, age, sector, socioeconomic score, obesity, Smoking status and Charlson score

### Patient experience

In the telephone survey 433 patients (or primary care giver) were contacted in the days following discharge from HAH,427 answered the questionnaire (98/6%). 84% of patients said they would prefer HAH over hospital admission for future care, however this fell to 77% amongst the Arab patients. 5% of respondents indicated a preference for receiving care within a hospital, while 11% expressed no preference. 90% of the patients said they felt they were "in good hands" when treated in HAH with no difference among Arab patients.

## Conclusions

### Summary of results

Numerous studies have been published on HAH; however, there remain questions over the safety, effectiveness, and scalability of HAH [2].

This large real-world study found that HAH patients had lower mortality and readmission rates at 30 and 90 days. Mortality was significantly reduced for acute infections like pneumonia, cellulitis, and UTI, but not for CHF. Readmission rates were also lower in the HAH group for pneumonia and UTI at both time points. Among patients with CHF, no statistically significant difference in readmission rates was observed. However, the direction of the effect suggested a trend toward increased readmissions at 90 days in the HaH group (OR = 1.33, p = 0.065).

### Comparison to known literature

A survey on attitudes to HAH in Israel found that 78% of the public and 97% of physicians consider HAH a good alternative to traditional hospitalization and would personally use it [31]. In the post-treatment telephone survey, 84% of respondents preferred HAH over hospital admission for future hospital-level care needs. Showing that HAH is widely acceptable to both Israeli medical professionals as well the public.

A study of homebound patients with a high burden of chronic disease, within the framework of a HAH service in southern Israel showed a reduction in costs and of hospital admissions [32]. According to the Israel Ministry of Health’s price list, one day in a general medical ward costs 2,900 NIS. During the study, HAH treatment cost less than half as much per day as hospital care, with similar admission durations. Fewer readmissions occurred with HAH, suggesting potential economic benefits over hospital care.

In a review article examining nine studies, providing data on 959 participants with exacerbation of chronic illnesses, mortality did not differ between HAH and the in-hospital care groups (RR, 0.84; 95% CI, 0.61–1.15), and risk of readmission was lower (RR, 0.74; 95% CI, 0.57–0.95) [25]. Similarly, in a review of CHF patients admitted to HAH compared to a hospital, HAH demonstrated a trend to decreased readmissions (risk ratio (RR) 0.68 [0.42 to 1.09]) and did not affect all-cause mortality (RR 0.94 [0.67 to 1.32]) 14[]. These results are similar to the current study.

In a trial of 91 patients divided randomly assigned to HAH or admitted to the hospital, HAH patients were less often readmitted within 30 days after discharge (7% vs. 23%) [11]. A Cochrane review for a mix of conditions reported a trend towards lower mortality under HAH schemes at 3 months (RR 0.77, 95% CI 0.54 to 1.09) and a significant reduction at 6 months (RR 0.77, 95% CI 0.60 to 0.99; moderate-quality evidence) [12]. In a further review no significant effects on mortality were found (RR 0.94; 95% CI 0.67 to 1.32) [33].

Previous studies imply, at least, non-inferiority for treatment in HAH. The current study adds further evidence to the safety and effectiveness of HAH when compared to traditional hospital care for patients with acute infectious illness or CHF exacerbations.

### Limitations

The main limitation of this study is its observational design and the possibility of selection bias. Patients were admitted to HAH only if they were hemodynamically stable, which introduces potential for selection bias. This bias could not be controlled by adjusting for acuity of illness at admission, as such data were unavailable. This factor may account for the higher mortality in the initial days following hospital admission compared to HAH. To address this issue, the following approaches were taken: 1. A two-stage analysis was performed, matching HAH to hospital patients, followed by multivariate logistic regression for relevant clinical factors. 2. Only patients admitted to general internal wards were included, with ICU admissions excluded. Given the observational nature of the study, it is likely that unmeasured biases remain, such as the inability to compare acuity of illness at admission, and these may result in an overestimation of the benefit of HAH. Nonetheless, the reported reduction in mortality rates by 40% and readmission rates by 20% indicates that HAH is both safe and effective.

The principal strength of the study lies in its real-world, nationwide design, which ensured a sufficiently large sample size to demonstrate the scalability of HAH studies.

### Policy implications

Our study demonstrates that a large HAH program with over 15,000 annual admissions delivers safe, effective care that most patients prefer to hospital admission. Given its lower cost compared to traditional hospital care and similar duration, we recommend HAH as the preferred option for medically eligible patients, subject to patient preference and adequate social support at home.

### Conclusions

HAH can provide a safe and effective environment to treat patients who need hospital-level care, with high levels of patient satisfaction. HAH has the potential to provide a scalable solution for the ever-increasing demand for hospital beds.

## Data Availability

The data for this article comes from Clalit Health Services data bank. To preserve patient privacy this data is not currently available for public access.

## Abbreviations

HAH: Hospital at home
CHS: Clalit Health Services
CHF: Congestive heart failure
IHD: Ischemic heart disease
SES: Socio-economic status
SSA: Small statistical areas
